# Implementing Semi-Automated Medication Dispensing for People with HIV: A Community-Based Alternative to Traditional Pharmacy Pickups

**DOI:** 10.3390/healthcare14040429

**Published:** 2026-02-09

**Authors:** Diana Hernández-Sánchez, Jorge Saz, Ignacio García Gimenez, Jordi Puig, Angel Rivero, Patricia Valero, Maria Isabel Martinez, Rafael Muñoz, Carles Quiñones, Meritxell Davins Riu, Eugenia Negredo

**Affiliations:** 1 Fundació Lluita Contra les Infeccions, 08216 Badalona, Spain; 2 Departamento de Medicina, Universitat Autònoma de Barcelona, 08193 Barcelona, Spain; 3 Projecte dels NOMS Hispanosida–BCN Checkpoint, 08015 Barcelona, Spain; 4 Departamento de Farmacia, Hospital Universitari Germans Trias i Pujol, 08216 Badalona, Spain; 5 Departamento de Enfermeria, Universitat Autònoma de Barcelona, 08193 Barcelona, Spain; 6 Servei de Malalties Infeccioses, Hospital Universitari Germans Trias i Pujol, 08216 Badalona, Spain; 7 Direcció Transformació i Salut Digital, Hospital Universitari Germans Trias i Pujol, 08216 Badalona, Spain; 8 Grup de Recerca INEDIT, Institut de Investigació Germans Trias i Pujol, 08216 Badalona, Spain; 9 Unidad de Proyectos, Hospital Universitario Germans Trias i Pujol, 08216 Badalona, Spain; 10 Direcció d’Organització i Sistemes d’Informació, Hospital Universitari Germans Trias i Pujol, 08216 Badalona, Spain; 11 Departamento de Farmacia, Hospital Universitari Dr Josep Trueta i Hospital Santa Caterina, 17007 Girona, Spain; 12 Universitat de Vic-Universitat Central de Catalunya (UVic-UCC), 08500 Vic, Spain; 13 Centro de Investigación Biomedica en Red Enfermedades Infecciosas (CIBERINFEC), 28029 Madrid, Spain

**Keywords:** electronic pharmacy, out-patient dispensing, automated lockers, mobile application, HIV infection, patient-centered care, community health, treatment adherence

## Abstract

**Introduction**: Maintaining adherence and access to antiretroviral treatment is basic for good management of people with HIV (PWH), while enhancing patient satisfaction. With the aim of shifting from drug-centered into patient-centered care and integrating care interventions into community settings, here we share an outpatient hospital pharmaceutical care implementation model for PWH. This model involves the delivery of medication through a community center, BCN-Checkpoint, using a proprietary app and coordinated with automated locker systems. **Methods**: During the pre-implementation phase the circuit was defined and seven steps were considered critical for successful implementation: (1) assignation of teams and roles; (2) adaptation of the self-developed app; (3) development of a patient journey map; (4) locker installation and system integration with data from the electronic records; (5) staff training; (6) review of data protection regulations; (7) simulation tests. A two-phase simulation—with fictitious users and with real ones—validated the system. The implementation phase included an initial pilot study, in which 46 patients were included in the project. **Results**: System uptake was high, with strong adherence to the dispensing pathway; only five discontinuations due to personal preferences or availability of alternative dispensing pathways. Several barriers to implementation emerged, primarily categorized into technical issues, process and operational challenges, coordination, and user-related difficulties. However, a communitarian setting, flexible attention times and protocols, and the strong intersectoral collaboration between specialists are believed to increase patient retention and overall satisfaction. **Conclusions**: The implementation of an outpatient dispensing hospital medication model using an app and automated locker systems is feasible, considering detail to procedures, timely adaptations, and staff training.

## 1. Introduction

The global landscape of HIV care has seen significant advancements, driven by ambitious targets. The objectives until 2020 guided per the Joint United Nations Programme on HIV/AIDS (UNAIDS) were to ensure that 90% of people with HIV (PWH) were diagnosed, 90% of them on antiretroviral treatment (ART), and 90% of them virally suppressed. These goals have since evolved to an even more ambitious 95-95-95 for 2025 [1]. Currently, with the advent of potent, convenient, and well-tolerated antiretroviral combinations available, and “Treat All” policies, HIV-related morbidity and mortality have dramatically decreased, thereby increasing life expectancy for PWH. Thus, ART has shifted to a chronic care model of disease management [2] and current UNAIDS goals for 2030 include a fourth 95 objective focused on quality-of-life improvement and zero discrimination [1,2,3].

Maintaining adherence to ART is basic for effective management of infection and risk prevention. For that reason, optimizing patient access to medication is crucial for enhancing patient satisfaction and improving adherence. This is particularly relevant when considering hospitals not situated in central urban areas, such as the case of the Germans Trias i Pujol University Hospital in Badalona (HUGTiP), where geographic proximity to healthcare services for some patients can be a challenge. Access to medication outside of the hospital setting is a patient-centered approach that ensures greater availability through extended opening hours and closer geographic proximity, not only improving the patient’s experience but also indirectly contributing to easing the demands on hospital pharmacy services.

Beyond geographic barriers, many people with HIV continue to face practical challenges such as repeated pharmacy visits, long waiting times, rigid opening hours, and stigma associated with hospital-based medication dispensing. These everyday obstacles can contribute to treatment fatigue and reduced engagement in care. Such barriers are frequently mentioned during routine clinical interactions, and similar challenges may affect patients attending other hospitals located outside central urban areas. With the aim of integrating care interventions into community settings, the present article shares the implementation of a new semi-automated pharmaceutical care model for PWH treated with outpatient dispensing hospital medication. This model involves the delivery of medication through BCN Checkpoint—a community-based center located in central Barcelona that focuses on HIV and sexually transmitted infections (STI) screening for men who have sex with men (MSM) and trans individuals—using a proprietary mobile application (app) and coordinated with automated locker systems. BCN Checkpoint plays a key community role in HIV prevention and linkage to care, which makes it a familiar, trusted, and highly accessible setting for our target population. Its pre-existing link with our center is an important facilitator in all implementation efforts.

## 2. Methods

This is an implementation/feasibility study of a novel, semi-automated pathway for PWH to access their outpatient dispensing hospital medication through self-service devices. Specifically, it evaluates the use of automated dispensing lockers as an alternative to traditional pharmacy dispensing. This process was mediated by eSalut360 MetroNord, a digital platform developed by Doole Health, building upon insights from a previous self-developed pilot application [4] that had inspired the approach. This involved a two-stage process.

### 2.1. Pre-Implementation Phase

The first stage was a pre-implementation phase, where (1) teams, roles and responsibilities were assigned among investigators and collaborators, encompassing clinical pharmacists, nursing staff, HIV and Information Technology (IT) specialists, and digital transformation experts from HUGTiP and BCN Checkpoint; (2) barriers and risks were assessed; (3) modifications were integrated in the app self-developed and technical adaptations took place; (4) a patient journey map was developed, illustrating each touchpoint from prescription at HUGTiP to medication retrieval at BCN Checkpoint; (5) automated dispensing lockers were selected by their features, physically installed at BCN Checkpoint, and their software was tuned to synchronize with selected dispensing data from the electronic records; (6) staff training sessions were conducted for all personnel involved, including staff from BCN Checkpoint and the hospital, to ensure proficiency in operating the new locker system and understanding the medication delivery protocols; and (7) a thorough review of data protection regulations was conducted. Once all these concerns were gathered in a procedures protocol, which encompassed standard operating procedures (SOPs) for every step of the automated dispensing process, a two-part simulation test was performed.

### 2.2. Implementation Phase

The second stage was an implementation phase. In an initial descriptive pilot study, 46 patients were included in the project. Participants were recruited consecutively among eligible patients (convenience sampling). As this was an exploratory feasibility pilot, no formal sample size calculation was performed; the sample size was determined pragmatically by operational capacity. The inclusion criteria were reviewed during the medical or pharmaceutical visits. Following selection, an electronic informed consent form had to be digitally signed up through the app, within a 7-day period. Inclusion criteria included PWH being on stable ART (>6 months), having a record of good adherence to ART and medication collection at the hospital pharmacy on time and agrees to collect medication every two months, and to be willing to use an app. Basic descriptive characteristics of the population and descriptive feasibility indicators (such as the number of medication pick-ups or cancelations) were collected from the clinical records, as well as extracted data from the Columat database spanning 2024 and 2025 periods, and analyzed using Microsoft Excel. This study has been conducted within a framework project that is the eSalut program. The project was approved through the Research Ethics Committee at Hospital Universitari Germans Trias i Pujol, Spain, with approval number PI-24-025, approval date 7 April 2024. All participants signed informed consent via the app, and no personal data was used.

During the first weeks of this phase, a video call was held the day before each medication delivery, involving a member of the pharmacy team, the Information Systems team, the Digital Transformation team, and the BCN Checkpoint team. The aim was to verify that all individuals who had requested medication through the app were correctly registered in SAP also appeared in Columat with a reserved locker. All barriers and facilitators of implementation emerging from these procedures were collected, agreed by all participants of the study, and the data that arrived at consensus is presented in this manuscript.

## 3. Results

### 3.1. Pre-Implementation Stage: Establishment of an Implementation Protocol

During 2023, prior to implementation, weekly meetings took place in which the leaders of the project discussed the strategy for successful implementation of the intervention. The group included a member from the technical staff for outpatient care, the pharmacy staff, the direction and secretary of the healthcare service, as well as physicians, nurses and non-clinical staff from HUGTiP and BCN Checkpoint. After a careful assessment of the available resources, it was agreed that the informatic platforms to be used for the implementation would be Silicon (for registry of drug disposals), SAP (to link the patient clinical records with the medical prescriptions) and Columat (owners of self-automated lockers equipped with personalized software). To ensure proper communication with the patient, the mobile app eSalut360 MetroNord, powered by Doole Health, was agreed to be employed. The eSalut360 MetroNord application was originally developed specifically for the needs of PWH. In relation to this study, the app includes a user-friendly calendar showing available pick-up time slots and allowing appointment changes, as well as optional notifications to support adherence to the dispensing pathway. It is fully integrated with SAP ARGOS for direct scheduling within the pharmacy’s agenda. Nevertheless, a pharmaceutical technician must document prescriptions and disposal dates on paper throughout the whole process to maintain traceability, as well as manage the agenda and send the medication. An external messenger was contracted for deliveries under the appropriate drug conservation conditions on a regular basis. Importantly, it was also agreed that the procedure would only be carried out with thermostable medication.

**Figure 1:**
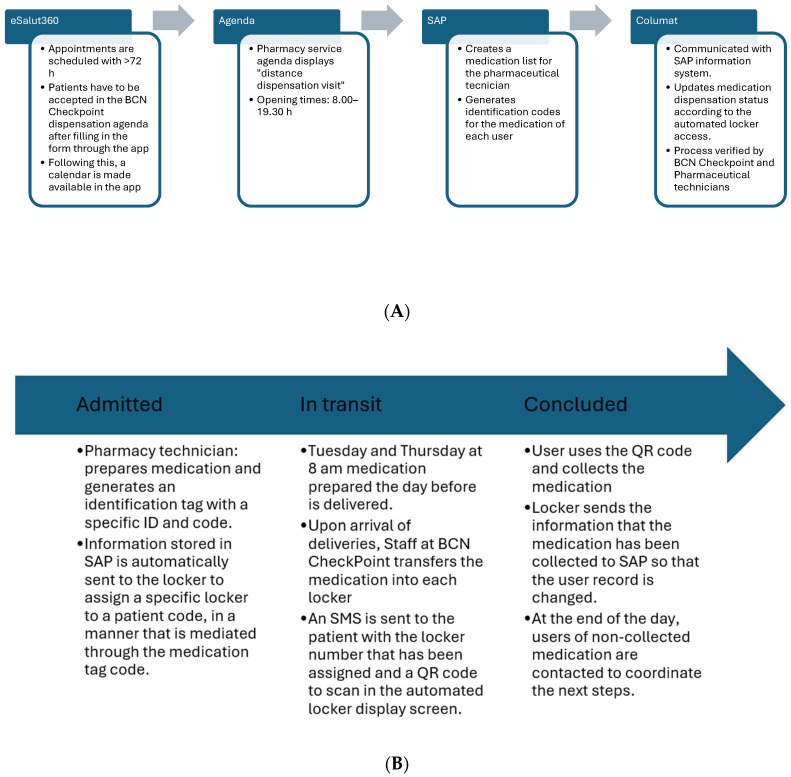
Information systems functions and flow (**A**), and coordination with staff/user interaction, as mediated by SAP status (**B**). In (**A**), data flow between the different information systems (Columat, SAP, eSalut360 MetroNord app) and their functions are described throughout the whole pathway allowing for acceptance into the program, creating appointments in the agenda, and providing traceability. In (**B**), the integration in the clinical records as mediated by SAP is described according to the stage of dispensing.

According to this, main teams with distinctive roles were distributed as follows: (1) pharmacy system, in charge of coordinating agendas, prescriptions, SAP registries, and shipping of medication; (2) clinicians, nursing and technician staff from BCN Checkpoint, in charge of user attention, appropriate handling of medication at the receiving center as well as coordination of non-collected medication; (3) information systems technicians and digital transformation specialist, in charge of the information network between Columat, SAP and the eSalut360 MetroNord app that supports a good flow of information between systems; and (4) clinician team from HUGTiP, which was in charge of including participants by supervising inclusion criteria, handling the initial information sheet (specifically designed with this purpose) to patients and register them in the eSalut360 MetroNord program, initiating the patient journey flow. Figure 1A,B illustrate how information systems (Columat, SAP, eSalut360 MetroNord app) were interconnected to ensure the information flow, in coordination with staff and user actions.

Regarding the planning of agenda, team leaders established a maximum daily patient pickup limit for lockers, determined by their capacity and availability. Appointments had to be scheduled at least 72 h in advance to facilitate necessary preparations and minimize delivery risks. The service was facilitated by being available according to the clinic opening hours, from 8 to 20, The number of days set for deliveries was optimized according to the demand, initially set off for two days per week and escalated to three days per week after successful implementation. The system is designed for further scalability, allowing for an increase to more daily delivery slots, or even multiple morning and afternoon shifts, shall demand continue to escalate. A description of the Patient Journey integrating these aspects is summarized in Table 1. In case of non-collection of the medication at the end of the day, staff from BCN Checkpoint retire it from the lockers, which switch the status on SAP to non-collected and initiates the circuit for the non-collected medication. This initiates with bidirectional communication between Checkpoint and Hospital Pharmacy to coordinate the return of medication. Additionally, an SMS reminder is sent that non-collected medication is no longer available for withdrawal from the lockers, and a need to request again an appointment through the usual channels at the Pharmacy Service of the Hospital.

At first, a test was conducted with four fictitious users to verify the proper functioning of the locker management application, the receipt of the order by the pharmacy department, and the correct updating of records in the Systems, Applications and Products in Data Processing (SAP) system, the information system to manage and monitor our patients. Subsequently, a second simulation test was carried out with four real users. This test made it possible to evaluate appointment scheduling through the mobile app, the medication loading process into the lockers, the reception of the Short Message Service (SMS) with the pickup code, and the correct performance of the remaining digital processes in both SAP and Columat.

### 3.2. Implementation Stage

#### 3.2.1. Pilot Implementation: Descriptive Statistics

A total of 46 patients were included in the pilot implementation—all men, with a mean age of 42.8 years (SD 9.03), 82.6% were from Barcelona and 17.4% from outside the city. According to registered data in the Columat registries, of 46 patients included, since November 2024, only five users have discontinued using the locker system. Follow-up in discontinuing users indicate mainly personal preferences or an alternative dispensing pathways (home delivery dispensing) as reasons for discontinuation, and not due to technical problems. The remaining users have been recurrently using the system every 2 months, as expected. During the whole period, the mean number of pickups has been of 8.5 (SD 1.78) per user—close to the expected maximum of 10—indicating strong adherence to the dispensing pathway evaluated despite initial procedural adjustments and minor technical issues. A total of five canceled services have been recorded. Duplicate entries were recorded on a total of four occasions.

#### 3.2.2. Barriers and Facilitators

During the pilot phases, several barriers to successful implementation emerged, primarily categorized into technical issues, process and operational challenges, and user-related difficulties. Addressing these issues was crucial for optimizing the system’s efficiency and user experience.

*Technical and system-related barriers*: Technical hurdles primarily revolved around the integrated information systems. A significant issue was the failure of SMS notifications, preventing users from receiving order confirmations and QR codes for locker pickup. This was often due to discrepancies in phone numbers registered in the SAP system, and the presence of foreign numbers unsupported by the system. In both scenarios, the solution involved updating the user’s contact information in SAP to a valid national mobile number. Another security-related technical barrier involved app accessibility; restrictions in certain countries prevented users from downloading the eSalut360 MetroNord application from their respective app stores. Furthermore, instances of duplicate medication entries in SAP occurred when requests submitted simultaneously appeared in the Columat platform. This issue was subsequently resolved. Finally, unreadable barcodes printed by the hospital pharmacy sometimes hindered automatic locker opening, requiring manual intervention to open the lockers by using the numeric code.

*Process and operational barriers*: Operational challenges also impacted efficiency. A key limitation in the user registration circuit was a system constraint requiring the entire process to be restarted if not completed within 7 days. Many users missed this deadline, likely due to the non-immediate need for medication collection and insufficient information regarding data processing delays. Additionally, while non-collected medication was updated in SAP, manual patient contact and subsequent delivery on the following day introduced extra administrative steps for traceability.

Given that the healthcare centers do not belong to the same city, the hospital (HUGTiP) being based in Badalona, and BCN Checkpoint in Barcelona, there might be emerging difficulties arising from this, such as differences in local festivities and opening days, that have to be considered when coordinating agendas.

*User-related barriers*: User-related difficulties centered on digital literacy and understanding of the system. Some users, particularly older individuals or those with limited digital proficiency, found navigating the app and requesting medication challenging. One of the main limitations was the process of signing the informed consent form through the app; patients either found the localization of the forms non-intuitive, or encountered limitations on form availability, which resulted in additional administrative hurdles for both parties. Clear instruction to patients in this regard is essential, as informed consent is necessary for these types of projects, and other facilitators might need to be considered. To mitigate these user-centric issues and enhance overall process understanding, detailed step-by-step instructions were integrated into the app, in-person assistance was provided by hospital and BCN Checkpoint staff, a dedicated email support channel was established, and thorough training was implemented across the clinical department. Despite these challenges, all affected users were ultimately able to receive their medication, demonstrating the adaptability of the Columat platform through features like manual locker opening.

## 4. Discussion

Unmet needs regarding access to healthcare resources and ART administration for PWH by hospital pharmacies have been previously described [5]. The traditional model of outpatient hospital medication dispensing often presents significant barriers to patient-centered care, particularly for individuals requiring long-term treatment such as PWH. These challenges include inflexible pick-up times, geographical limitations (long travel times, limited public transport options, parking difficulties and the pickup being overall time consuming), and a lack of privacy, which can collectively impact on treatment adherence and overall quality of life. Such limitations are frequently mentioned during routine clinical interactions, and similar challenges may affect patients attending other hospitals located outside central urban areas. These observations further reinforce the relevance of developing more accessible, community-based medication distribution models.

Crucially, these logistical hurdles are often exacerbated by the profound and persistent issue of HIV-related stigma. This is a complex issue [4] that has a detrimental impact on a variety of health-related outcomes such as delayed healthcare seeking, reduced access to services, and decreased medication adherence due to fear of disclosure or judgment [6]. In fact, about one fourth of PWH may be experiencing stigmatization in the healthcare setting [4]. Although we have not directly measured rates of anticipated stigma in our unit, recent survey data in Spain [7] indicated that, of 525 PWH questionnaire respondents, nearly half of them experienced some form of anticipated stigma. This type of stigma indirectly affects adherence to antiretroviral therapy, reinforcing the need to address its impact on the health of PLWH, as previously discussed in existing literature [8]. In line with this, community services provide a safer and closer environment for PWH communities, which might make these strategies particularly successful [9,10], likely translating into higher patient engagement, treatment adherence and psychosocial improvements. In fact, informal anecdotal non-systematic patient feedback obtained during implementation was consistently very positive However, one limitation of our study is the lack of a direct assessment of patient satisfaction, usability, or perceived benefit in order to compare with the traditional dispensing pathway.

Delivery of ART outside of traditional hospital settings may represent a key focus in HIV care. In this regard, the use of automated dispensing machines or lockers, as implemented in our study, represents a growing area of innovation in ART delivery. For instance, recent data from Eswatini, Africa, have demonstrated the feasibility and benefits of implementing Automated Medication Dispensing Systems to improve medication access and convenience for PWH, particularly as a component of differentiated service delivery [11]. As well, an automated Distribution System has been implemented by the Zambia government in a very large population with great outcomes in terms of adherence to ART [12]. Nevertheless, its broad implementation remains limited to a few cases [13]. In response, our project created and implemented a novel pharmaceutical care model designed to integrate medication delivery into accessible community settings. This innovative model leverages automated locker systems and a dedicated mobile app (eSalut360 MetroNord) to provide PWH with a convenient, discreet, and flexible alternative to traditional pharmacy pick-ups.

Our pre-implementation phase meticulously addressed various critical aspects to ensure a robust and seamless transition. This involved a two-part simulation test, and a comprehensive assessment of barriers and risks. The selection and installation of automated dispensing lockers at BCN-Checkpoint in central Barcelona underscored our commitment to community-integrated care. This location was strategically chosen to offer a familiar and accessible environment for our target population, reducing the stigma often associated with hospital visits. Community centers, such as BCN Checkpoint, play a critical role in connecting hospital services with the reality of PWH, particularly key populations such as MSM and transgender people. By locating ART dispensing in a trusted, stigma-free environment where people already have frequent STI testing, we believe this model strengthens engagement and encourages continuity of care. In addition to improving convenience and reducing the burden on hospital pharmacies, community centers offer a unique setting where medical, pharmaceutical, and psychosocial support can be integrated, thereby reinforcing treatment adherence and addressing the broader determinants of health [14,15]. This highlights the importance of leveraging existing community infrastructures as allies in innovative HIV care models. Furthermore, rigorous staff training and a thorough review of data protection regulations ensured operational readiness and patient privacy, with all data handling adhering to current data protection laws and no security breaches identified to date.

During the initial pilot study, we successfully enrolled 46 patients across three rounds. This phased inclusion strategy allowed us to progressively integrate users and address emerging issues. Performance metrics indicate good system uptake and continuity. Six months after inclusion, only five of the 46 enrolled patients had discontinued use, while the remaining participants continued collecting their medication every two months as expected. Users completed a mean of 8.5 pickups (SD 1.78), with only five canceled services. Considering that an optimal two-monthly schedule would allow for up to 10 pickups during this period, these results reflect strong adherence to the dispensing system despite initial procedural adjustments and the technical limitations described. However, limitations include the real world (non-controlled) nature of the follow up, as well as the initial system adaptations and technical issues that may have affected the number and regularity of pick-ups. As this is an implementation study, the absence of comparative or evaluative data is an inherent limitation. Nonetheless, anecdotal non-systematic feedback from patients, BCN Checkpoint staff, and the pharmacy team indicates a generally positive experience with the model.

As facilitators of implementation, the agreement to utilize existing informatic platforms such as Silicon (for drug disposals), SAP (for linking clinical records and prescriptions), and Columat (the self-automated lockers with personalized software) alongside the eSalut360 MetroNord app demonstrated a strategic use of available resources. The defined roles for pharmacy staff, BCN Checkpoint personnel, information systems technicians, and the hospital’s clinical team ensured a coordinated effort throughout the implementation. The agreed-upon operational parameters, such as a maximum of 50 patients per day for locker access, deliveries on two non-consecutive days, and a 72 h appointment anticipation, reflect a balanced approach to demand and timely medication. While these decisions were based on our needs at the moment, the parameters might need to be adapted to the context. For instance, the collection period might need to be updated, extending the period to 7 days as was implemented in other models [11]. Importantly, the focus on thermostable medication simplifies the logistics of external messenger services, ensuring appropriate drug conservation conditions. However, if future needs dictate, the system can be adapted to accommodate refrigerated medications given that to our knowledge there are already existing temperature-controlling lockers, such as Locktec Smart Lockers [16].

Despite the careful planning, the pilot phase revealed several practical challenges, providing valuable insights for continuous improvement. Issues appeared, such as the failure to receive SMS notifications due to incorrect or foreign phone numbers in SAP. These highlighted the critical need for accurate and up-to-date patient contact information, especially in a system reliant on digital communication. The resolution, which involved updating contact information and utilizing manual locker opening as a fallback, ensured that no patient was denied access to their medication. Duplicate requests in lockers and barcode printing errors were identified as areas requiring technical refinement and improved internal processes within the pharmacy. Restrictions on app downloads in certain countries appeared due to security protocols, which underscored the need for alternative access methods or geographically tailored solutions for international patients. Finally, addressing user difficulties with the app, particularly for older individuals or those with limited digital literacy, led to the implementation of step-by-step instructions and in-person assistance at BCN Checkpoint. This underscores the importance of digital inclusion in patient-centered care.

Although this model requires initial coordination and technological setup, once established, workload decreases, as pharmacy visits are reduced and processes streamline. However, the need for interoperable platforms, lockers, and trained staff may challenge centers limited in resources. Simplified versions or a phased implementation may improve feasibility, and long-term sustainability will depend on balancing initial costs with reduced pharmacy burden and improved patient flow. Nevertheless, benefits of implementing this model, given its structure may outcast its initial efforts, as it can be adapted for other chronic conditions that require regular medication collection, particularly where decentralizing pharmacy access may enhance adherence and reduce service burden.

While we have not analyzed specific data on user satisfaction, we anticipate positive results given our efforts to increase accessibility and streamline the medication collection process. This is further supported by existing evidence of increased user satisfaction in automated medication dispensing systems compared to the traditional pathway [17,18]. This expectation is also supported by the limited, but encouraging, published data on patient satisfaction with automated self-dispensing lockers [19]. Despite the acknowledged barriers and limitations, particularly concerning the significant human and economic resources required for implementation, the advantages of this system are substantial. A clear indication of enhanced patient experience is the growing adoption of the system in our center. As of August 2025, a total of 251 patients are active users of the eSalut360 MetroNord app, and 180 are actively using the locker-based medication dispensing system.

## 5. Conclusions

In summary, this successful project demonstrates the feasibility and potential of integrating community-based, semi-automated medication dispensing models into broader HIV care strategies. By leveraging community infrastructure, digital tools, and robust intersectoral collaboration, the project simplified the patient pathway and aligned care delivery with the evolving UNAIDS focus on quality of life. Although direct comparisons of adherence and retention measures to the standard delivery have not been made, this decentralized, patient-friendly approach not only enhances convenience but also holds promise for improving treatment adherence by removing traditional logistical barriers. Insights gained during the pilot phase are essential for scaling up the service and ensuring its long-term sustainability. However, the lack of direct satisfaction measures and long-term adherence to treatment outcomes limits the strength of these findings, highlighting the need for future studies to formally evaluate the long-term clinical impact and user experience of this model.

## Figures and Tables

**Table 1:** Patient Journey. Summarizes all the steps from the inclusion in the program, through the app mediated dispensing pathway, to the final medication collection.

Step	Description
1	The patient is informed during the medical visit about the delivery of medication through BCN Checkpoint, using a proprietary app and coordinated with automated locker systems
2	After consenting, the doctor signs up the user on the eSalut360 MetroNord system through an access on SAP, that links the clinical history to the app.
3	A link is sent to the patient e-mail to create an account for the app.
4	Two subsequent consent forms are sent through the app, that are validated by the IT team and the pharmacy team, which enable a calendar to book deliveries of the medication. This calendar is synchronized with SAP.
5	After the patient books a specific date, the pharmacy technician in charge will prepare the medication according to the BCN Checkpoint agenda which is scheduled on SAP
6	When medication is prepared the patient status on SAP will change, which in turn sends information to the locker system in a manner that is linked to a unipersonal code printed on the patient label.
7	The preparation will be in specific transport boxes for BCN CheckPoint, by date of collection and with delivery notes to the suitcase and to the destination center.
8	Boxes are sent from the pharmacy service at a fixed time of 7 am, with arrival at 8 am. Sending is registered and delivery notes kept for records.
9	When the auxiliary or nursing staff of the receiving center introduces the medication into the locker, the status will be changed to ‘in progress’ and an SMS will be sent to the patient with a QR code so that they can open the assigned locker.
10	When the patient picks up the medication, the locker will send information to the SAP to update the status to “concluded”. This system always ensures traceability of drug delivery.
11	Non-collected medication will be gathered at the end of the day at BCN Checkpoint using the QR code, so that users receive a message on their mobile phones with instructions to follow.

## Data Availability

The data that support the findings of this study are available on request from the corresponding author. The data is not publicly available due to privacy restrictions.
